# Supplementary material for: Nickel-organo compounds as potential enzyme precursors under simulated early Earth conditions
Source: Commun Chem. 2024 Feb 15;7:33. doi: 10.1038/s42004-024-01119-0 (PMC10869729; doi:10.1038/s42004-024-01119-0)
Supplement: Supplementary file 2 — Supplementary Information [file 42004_2024_1119_MOESM2_ESM.docx]

**Supplement**

**Nickel-organo compounds as potential enzyme precursors under simulated Early Earth conditions**

Philippe Diederich ^a^, Christian Seitz ^b^, Lance Buckett ^a^, Liesa Salzer ^a^, Thomas Geisberger ^b^, Wolfgang Eisenreich ^b^, Claudia Huber ^b^, Philippe Schmitt-Kopplin ^a, c, d*^

^a^ Helmholtz Munich, Research Unit Analytical BioGeoChemistry, Neuherberg, Germany.

^b^ Technical University of Munich, TUM School of Natural Sciences, Department of Bioscience, Bavarian NMR Center (BNMRZ), Structural Membrane Biochemistry, Lichtenbergstr. 4, 85748 Garching, Germany

^c^ Comprehensive Foodomics Platform, Chair of Analytical Food Chemistry, TUM School of Life Sciences, Technical University of Munich, Maximus-von-Imhof-Forum 2, 85354 Freising, Germany

^d^ Max Planck Institute for Extraterrestrial Physics, Center for Astrochemical Studies, Gießebachstraße 1, 85748 Garching bei München, Germany.

^*^Corresponding author: Research Unit Analytical BioGeoChemistry, Helmholtz Zentrum München–German Research Center for Environmental Health, Neuherberg, Germany.

Email: schmitt-kopplin@helmholtz-muenchen.de

**Color correlates with the bis(dithiolene)nickel signal**

The color of the analyzed samples correlates with the intensity of the mass of nickel bis(dithiolene). The bar plot presents the signal intensity at different time points. The corresponding sample is located directly beneath the respective bar and displays the reported color.


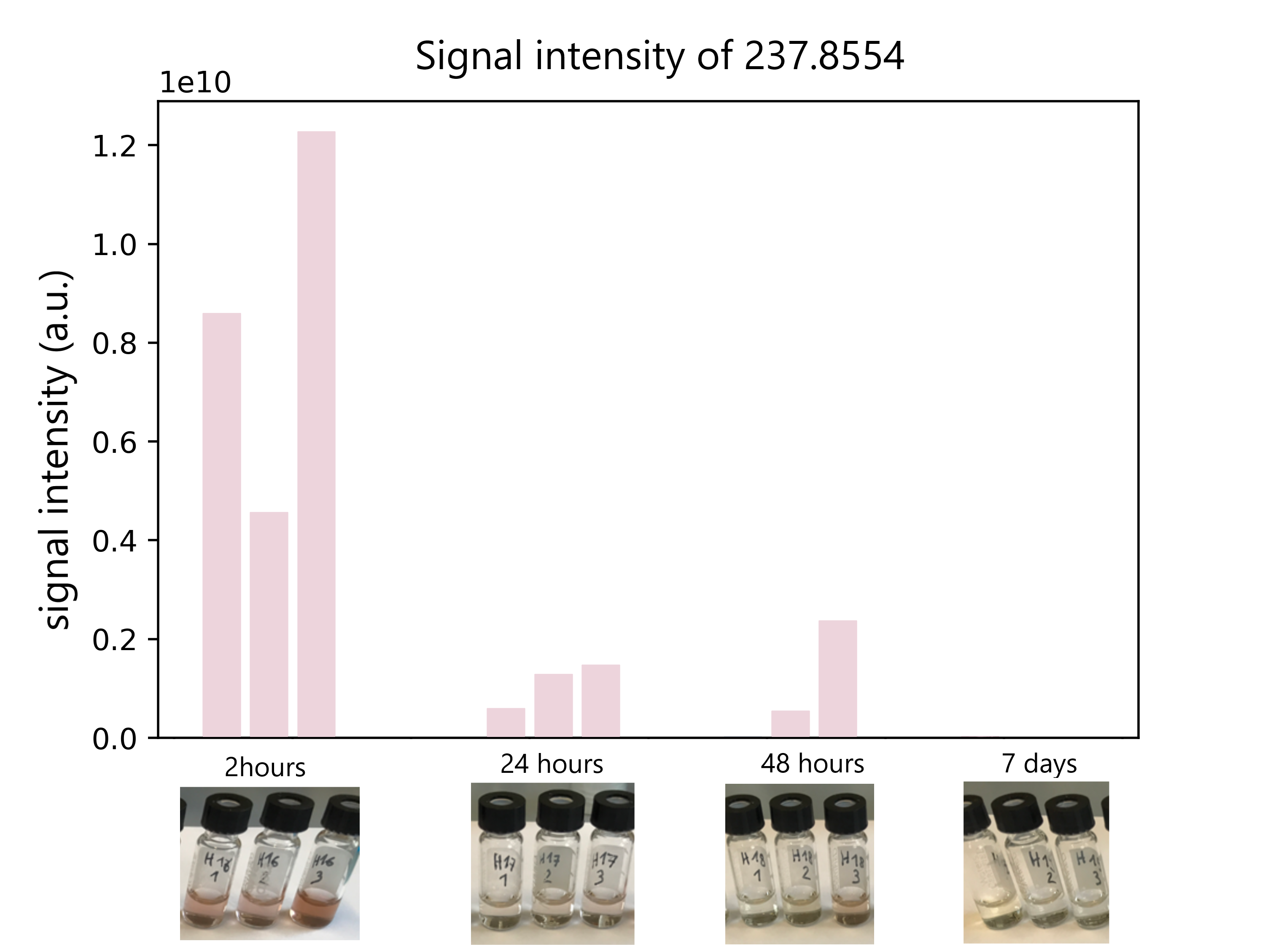


Supplementary figure 1: Bar plot showing the correlation between the detected intensity for the mass 237.8554 and the pinkish color of the prepared samples at different timepoints.

**Reaction scheme and discussed compounds**

**
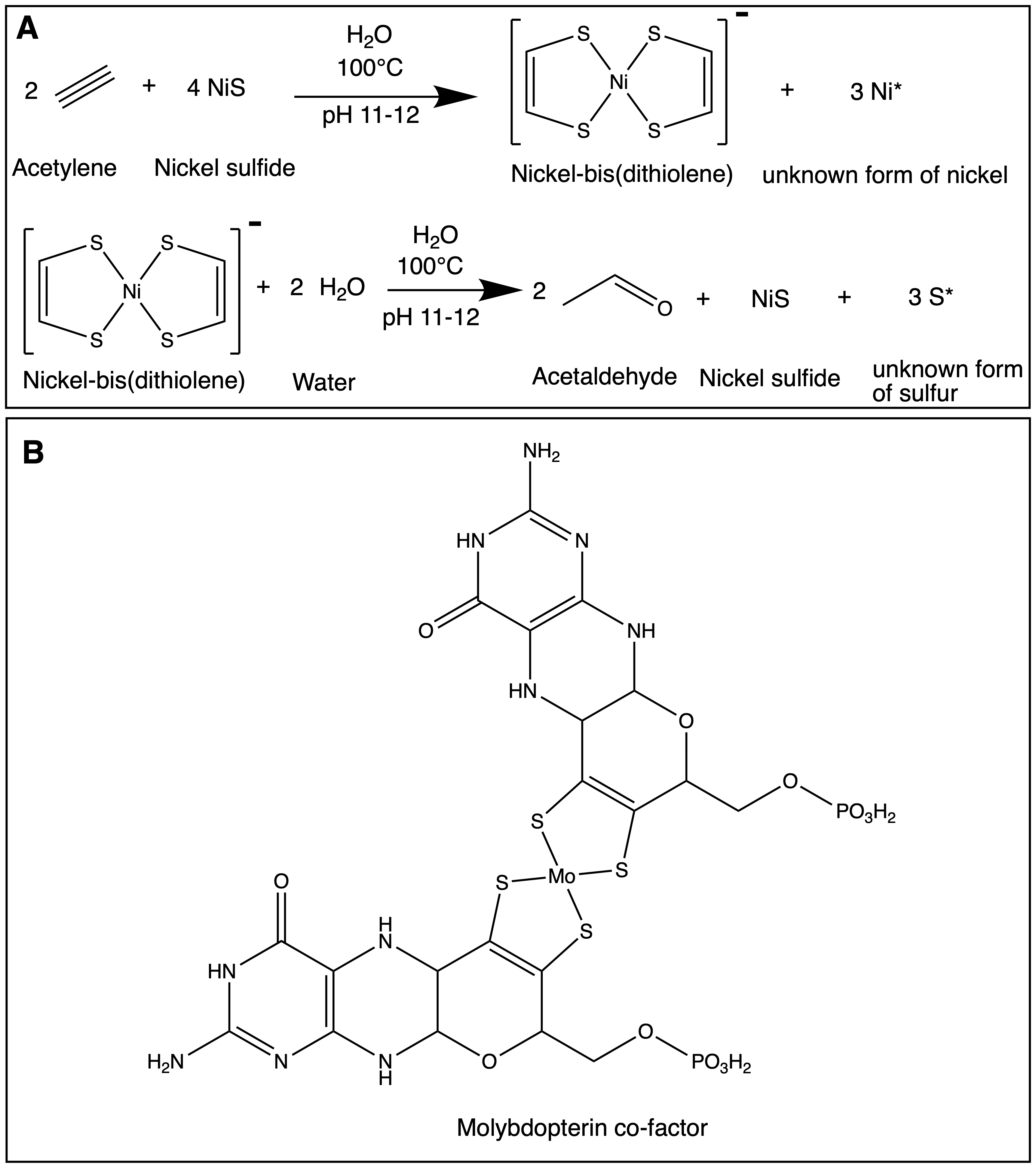
**

Supplementary figure 2: Reaction scheme overview. Panel A shows the hypothesized formation pathway of nickel bis(dithiolene) and acetaldehyde. Panel B shows the structure of a molybdopterin co-factor found in extant enzymes. *Unknown form.
