# Supplementary material for: Nickel-organo compounds as potential enzyme precursors under simulated early Earth conditions
Source: Commun Chem. 2024 Feb 15;7:33. doi: 10.1038/s42004-024-01119-0 (PMC10869729; doi:10.1038/s42004-024-01119-0)
Supplement: Supplementary file 3 — Description of Additional Supplementary Files [file 42004_2024_1119_MOESM3_ESM.pdf]

Description of Additional Supplementary Files

File name: Supplementary Data 1

Description: NMR spectra

File name: Supplementary Data 1

Description: Source data for figure 4
