# Supplementary material for: Nickel-organo compounds as potential enzyme precursors under simulated early Earth conditions
Source: Commun Chem. 2024 Feb 15;7:33. doi: 10.1038/s42004-024-01119-0 (PMC10869729; doi:10.1038/s42004-024-01119-0)
Supplement: Supplementary file 4 — Supplementary Data 1 [file 42004_2024_1119_MOESM4_ESM.pdf]

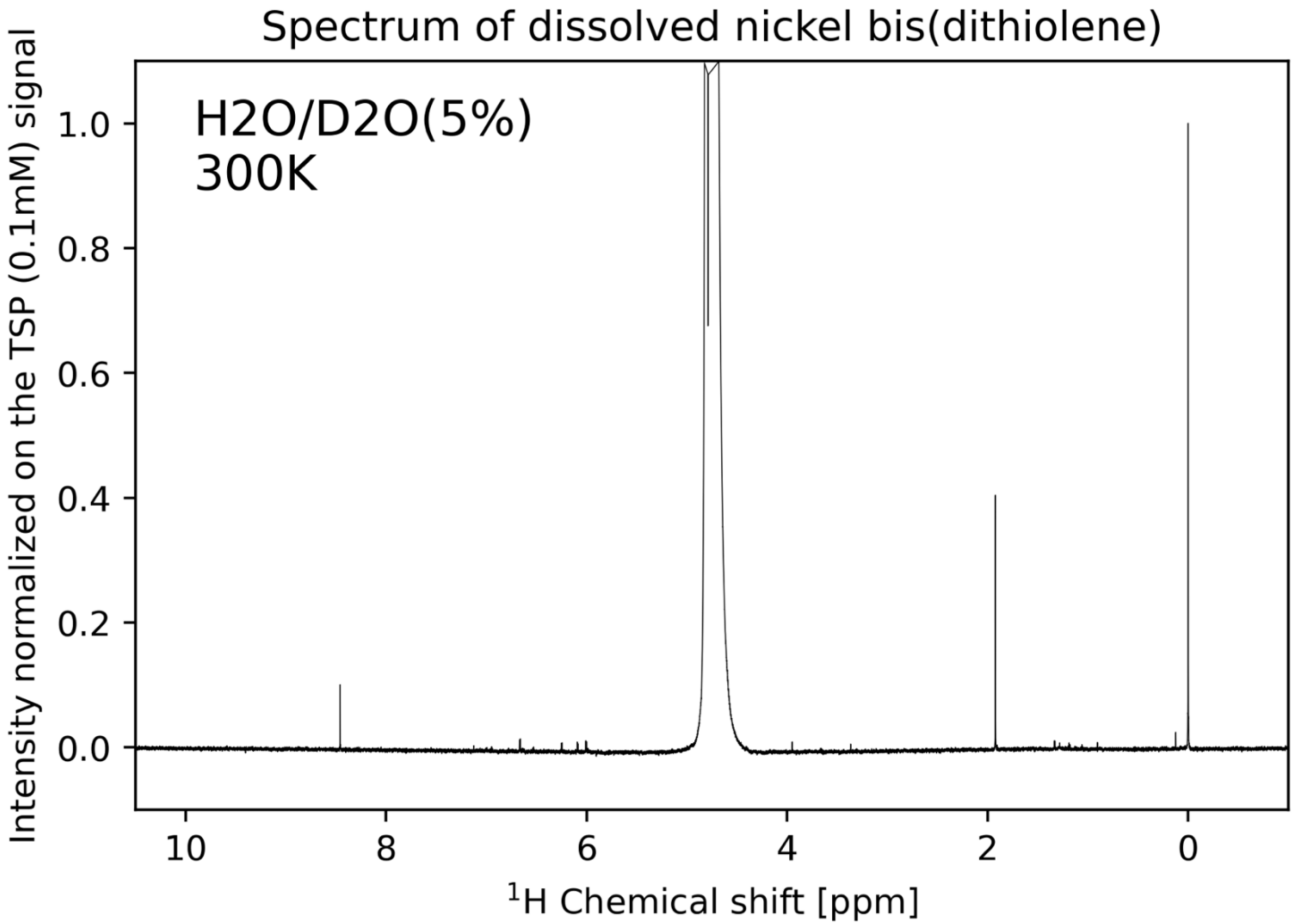

Spectrum of dissolved nickel bis(dithiolene)
H2O/D2O(5%)
300K
Intensity normalized on the TSP (0.1mM) signal
1.0
0.8
0.6
0.4
0.2
0.0
10
8
6
4
2
0
1H Chemical shift [ppm]

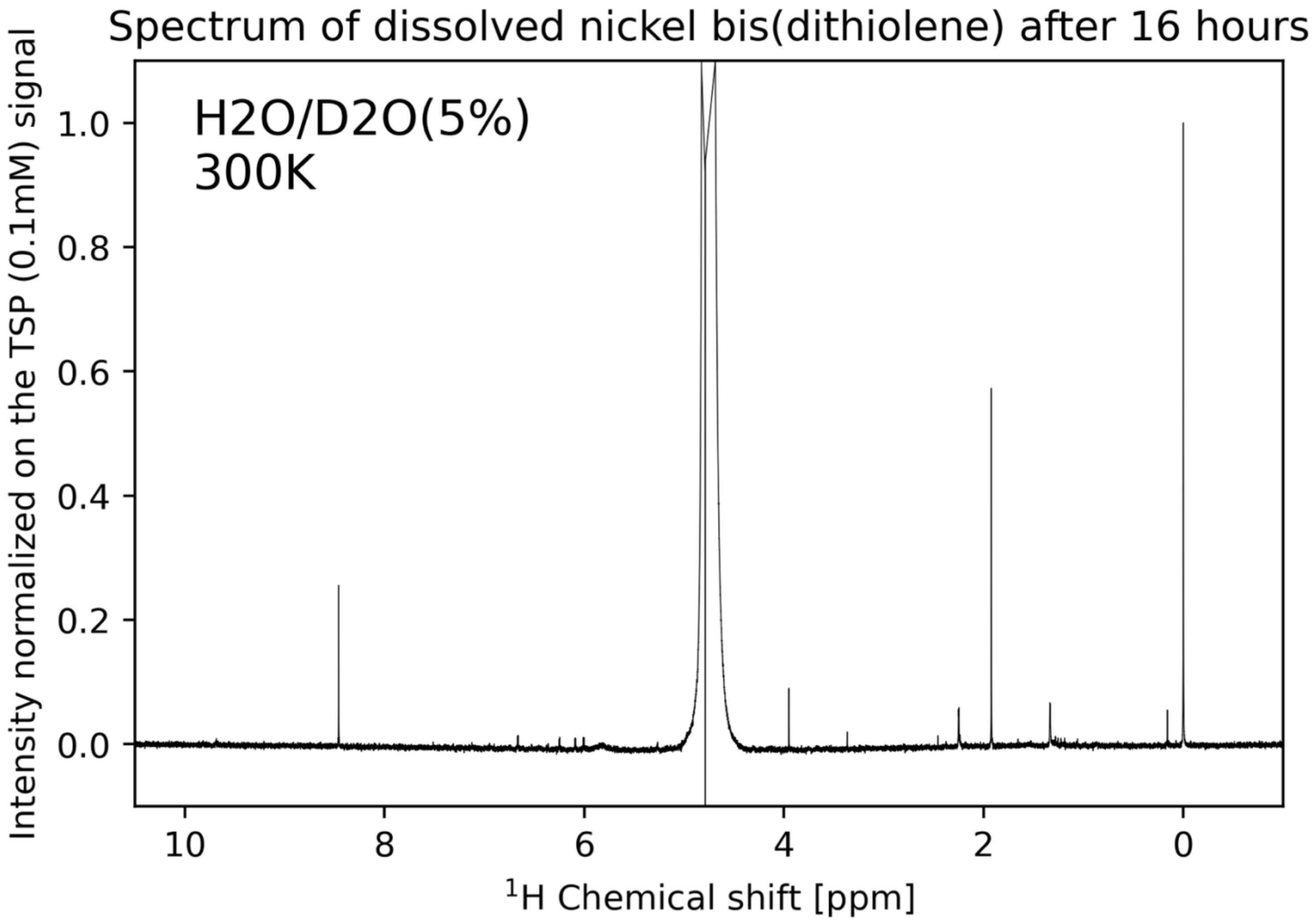

Spectrum of dissolved nickel bis(dithiolene) after 16 hours
H2O/D2O(5%)
300K
Intensity normalized on the TSP (0.1mM) signal
1.0
0.8
0.6
0.4
0.2
0.0
10
8
6
4
2
0
1H Chemical shift [ppm]

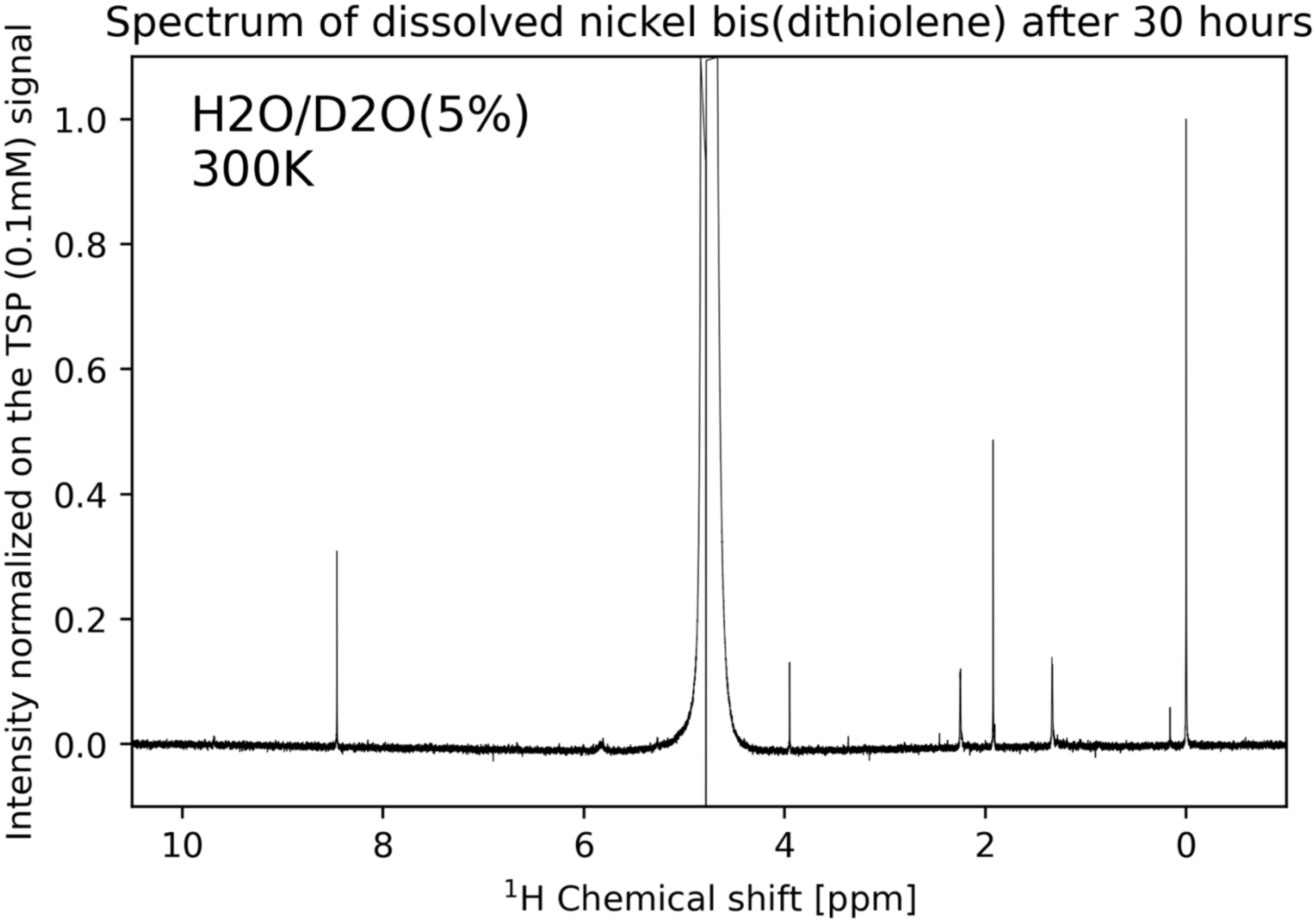

Spectrum of dissolved nickel bis(dithiolene) after 30 hours
H2O/D2O(5%)
300K
Intensity normalized on the TSP (0.1mM) signal
1.0
0.8
0.6
0.4
0.2
0.0
10
8
6
4
2
0
1H Chemical shift [ppm]

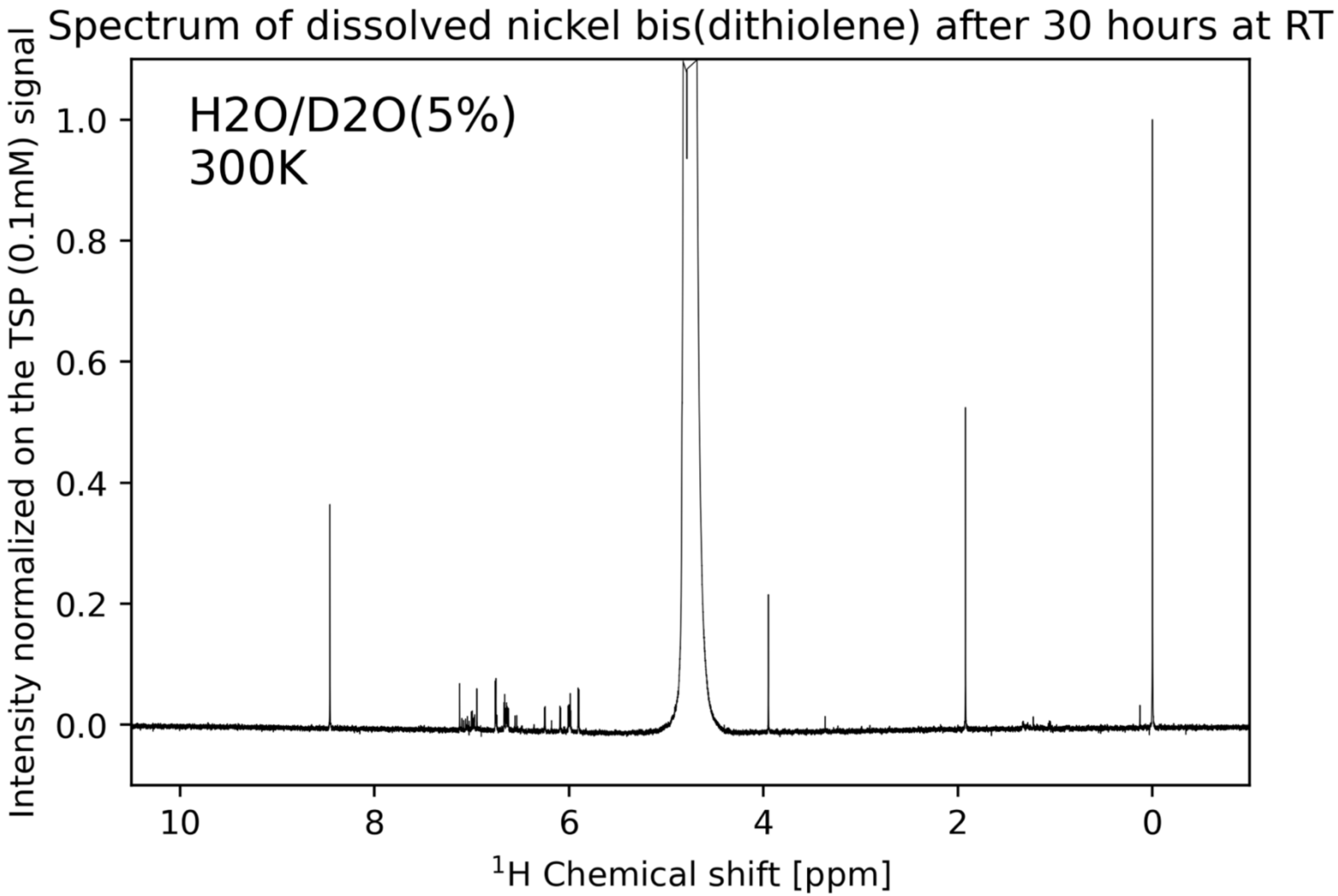
Spectrum of dissolved nickel bis(dithiolene) after 30 hours at RT
H2O/D2O(5%)
300K
Intensity normalized on the TSP (0.1mM) signal
1H Chemical shift [ppm]
1.0
0.8
0.6
0.4
0.2
0.0
10
8
6
4
2
0
